# Implementing and optimizing a communication curriculum in medical teaching: Stakeholders’ perspectives

**DOI:** 10.1371/journal.pone.0263380

**Published:** 2022-02-07

**Authors:** Christian Andreas Brünahl, Barbara Hinding, Leonie Eilers, Jennifer Höck, Anke Hollinderbäumer, Holger Buggenhagen, Kirsten Reschke, Jobst-Hendrik Schultz, Jana Jünger

**Affiliations:** 1 The German National Institute for state examinations in Medicine, Pharmacy and Psychotherapy (IMPP), Mainz, Germany; 2 Institute and Outpatients Clinic for Psychosomatic Medicine and Psychotherapy, University Medical Center Hamburg-Eppendorf, Hamburg, Germany; 3 Rudolf Frey Lernklinik, University Medical Center of the Johannes Gutenberg-University Mainz, Mainz, Germany; 4 University Clinic for Nephrology and Hypertension, Diabetology and Endocrinology, University Hospital Magdeburg, Magdeburg, Germany; 5 Department of General Internal Medicine and Psychosomatics, Heidelberg University Hospital, Heidelberg, Germany; Universitat Luzern, SWITZERLAND

## Abstract

**Objective:**

The relevance of communication in medical education is continuously increasing. At the Medical Faculty of Hamburg, the communication curriculum was further developed and optimized during this project. This article aims to describe the stakeholders’ perceived challenges and supporting factors in the implementation and optimization processes.

**Methods:**

The initial communication curriculum and its development after a one-year optimization process were assessed with a curricular mapping. A SWOT analysis and group discussions were carried out to provide information on the need for optimization and on challenges the different stakeholders faced.

**Results:**

The curricular mapping showed that the communication curriculum is comprehensive, coherent, integrated and longitudinal. In both the implementation and the project-related optimization processes, support from the dean, cooperation among all stakeholders and structural prerequisites were deemed the most critical factors for successfully integrating communication content into the curriculum.

**Conclusion:**

The initiative and support of all stakeholders, including the dean, teachers and students, were crucial for the project’s success.

**Practice implications:**

Although the implementation of a communication curriculum is recommended for all medical faculties, their actual implementation processes may differ. In a “top-down” and “bottom-up” approach, all stakeholders should be continuously involved in the process to ensure successful integration.

## 1. Introduction

Communication competencies are highly relevant in daily communication between doctors and patients and among hospital staff. Research has shown that good medical communication skills positively impact patients’ well-being, satisfaction, cooperation and health [1–7]. Furthermore, good communication skills among physicians were associated with those physicians’ increased well-being and job satisfaction [1–4]. Inefficient communication thus presents a threat to patient safety and health system resources [5, 6]. Therefore, it is crucial to teach and test relevant competencies in medical schools, as they are responsible for providing students with the necessary skills and preparing them for their working life [7, 8].

In recent years, different policies have highlighted the importance of communication in medical studies in Germany [9]. In 2012, the first amendment to doctors’ licensing regulations laid the foundation for integrating medical communication skills into medical students’ training and national licensing examinations [10]. Simultaneously, the popularity of so-called model study programs for medical education, which has enabled innovative developments, has increased. The publication of the National Competence-Based Learning Objectives Catalogue for Medicine (NKLM) followed in 2015, creating a uniform basis for faculty members to further develop their curricula. The catalog defines the required competencies of future doctors and learning objectives and thereby ensures the quality of medical studies across the country. The NKLM includes 116 communication-oriented learning objectives in the following six competence areas: (1) concepts, models and general basics, (2) medical communication skills and tasks, (3) emotionally challenging situations, (4) challenging contexts, (5) sociodemographic and socioeconomic influencing factors, and (6) other media channels and settings [11–13]. However, the NKLM’s nonbinding character and the varying study and examination regulations at medical faculties result in different degrees of integration of communication in the curricula. Therefore, a fully integrated and longitudinal model communication curriculum was developed in the LongKomm project to support the implementation and integration of communication-oriented learning content in medical faculty curricula [14–19]. Moreover, the model communication curriculum recommends the number of teaching units (one teaching unit equals 45 minutes) within the NKLM’s communication-related competence areas.

However, the implementation and factors that support the successful implementation of communication curricula have not yet been sufficiently researched. Consequently, in a multicenter study, the model communication curriculum was implemented at the Medical Faculties of Hamburg, Heidelberg, Mainz, and Magdeburg with financial support from the German Ministry for Health [20]. Information on faculty’s different baseline conditions was collected during a previous project [21–24]. The present research project focuses on the communication curriculum of the integrated model study program of the Medical Faculty of the University of Hamburg [25]. The program was introduced in 2012 and offers an innovative approach to the training of future doctors. One of the main differences to traditional medical study programs is that clinical and practical aspects are linked with theoretical subjects from the beginning of the six-year program. Additionally, medical skills in clinical examination methods and communication are provided from the first to the ninth semester through an interdisciplinary coordination platform.

The present study aims to describe stakeholders’ perspectives on the curriculum. Therefore, the degree of implementation of the communication curriculum at baseline and after an optimization process is quantified, which was carried out on the basis of the results of the baseline measurement. Furthermore, the factors that enable the successful implementation of longitudinal communication curricula and the strengths and weaknesses of the curricula will be identified.

## 2. Methods

### 2.1 Study design

**Fig 1:**
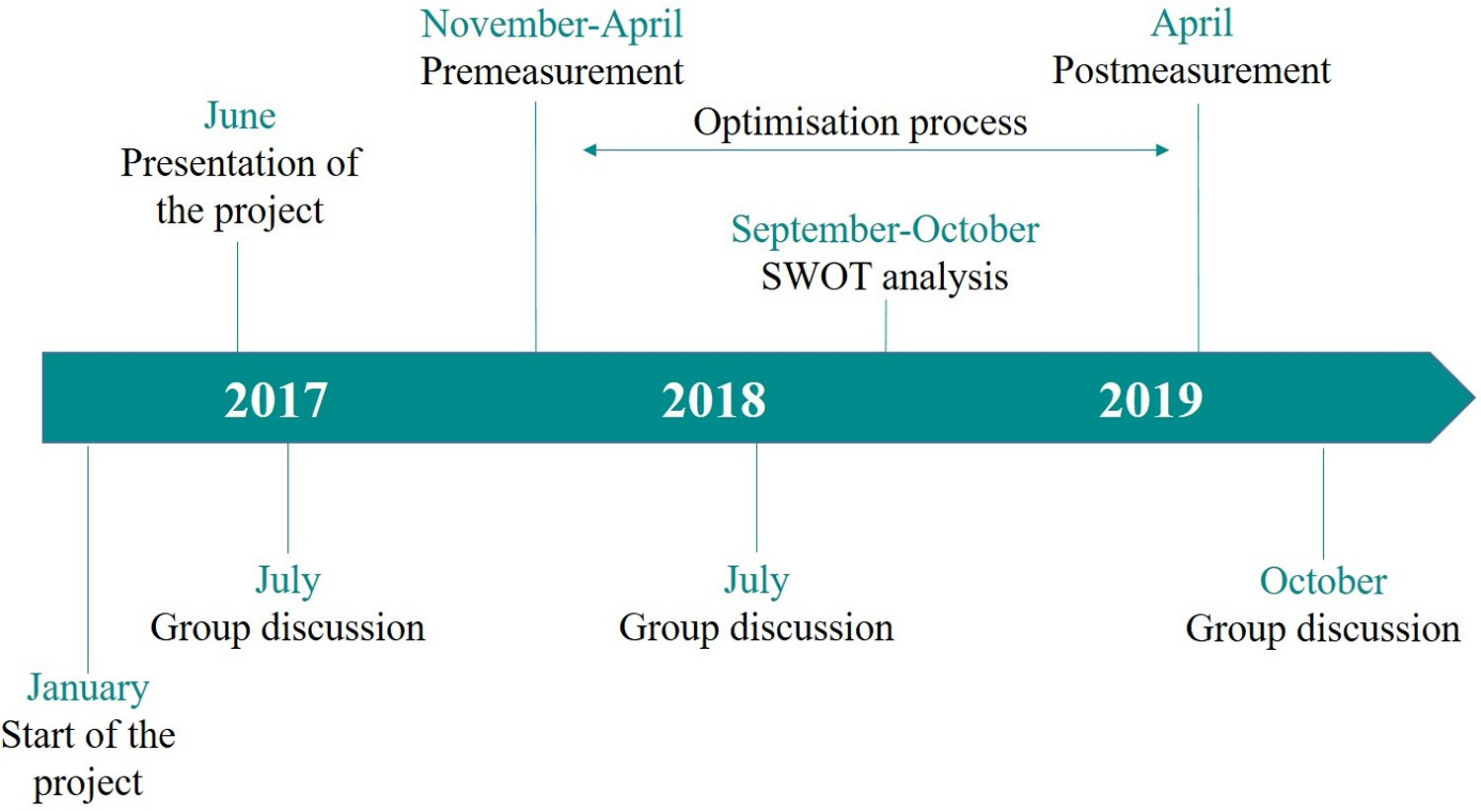
Study design and timeline. Description: The timeline shows the individual components of the project. Beginning in January 2017, the project was presented at the Medical Faculty of Hamburg’s annual faculty meeting in June. Above the timeline, the time points of the quantitative surveys and the SWOT analysis are shown. The optimization process took place between the pre- and postmeasurement. Group discussions took place in July 2017, July 2018 and October 2019.

Within the framework of a design-based research project, we involved both quantitative and qualitative methods [20, 26, 27]. As shown in Fig 1, the project started in 2017 and consisted of pre- and postmeasurements, three group discussions, a SWOT analysis and an optimization process. In June 2017, the project was presented at the annual teaching conference at the Medical Faculty of Hamburg to make the project known to a large proportion of the faculties’ staff. The aim of the first survey in 2017/18 (premeasurement) was to determine the current status of communication content within the curriculum, particularly in terms of courses with communication-related content, the number of teaching units (one teaching unit equals 45 minutes) and the coverage of learning objectives according to the NKLM. Based on the results, a cross-curricular steering group formulated goals and developed a strategy for the optimization process. However, this process was organized by the respective faculties and is beyond the scope of this paper. The optimization process concerned the consolidation and reduction of redundancies, derivation of further changes, and optimization of the curriculum and the framework conditions. Further, it included guidelines for teaching communication and hosting regular meetings of lecturers, information events, and meetings between disciplines and lecturers for fine-tuning the process. One aim of the project was to restructure the curriculum to reduce redundancies in favor of less well-covered learning objectives. A second survey was carried out in 2019 (postmeasurement), and individual and group interviews were conducted during the project.

Although the study scope exempted it from German requirements for formal ethical review by an institutional review board, all procedures performed in this study involving human participants were in accordance with the ethical standards of the institutions and with the WMA Declaration of Helsinki. Prior, written informed consent was obtained from the individual participants involved in the curricular mapping. Verbal informed consent was obtained from all participants involved in the voluntary interviews.

### 2.2 Quantitative method (pre- and postmeasurement)

**Fig 2:**
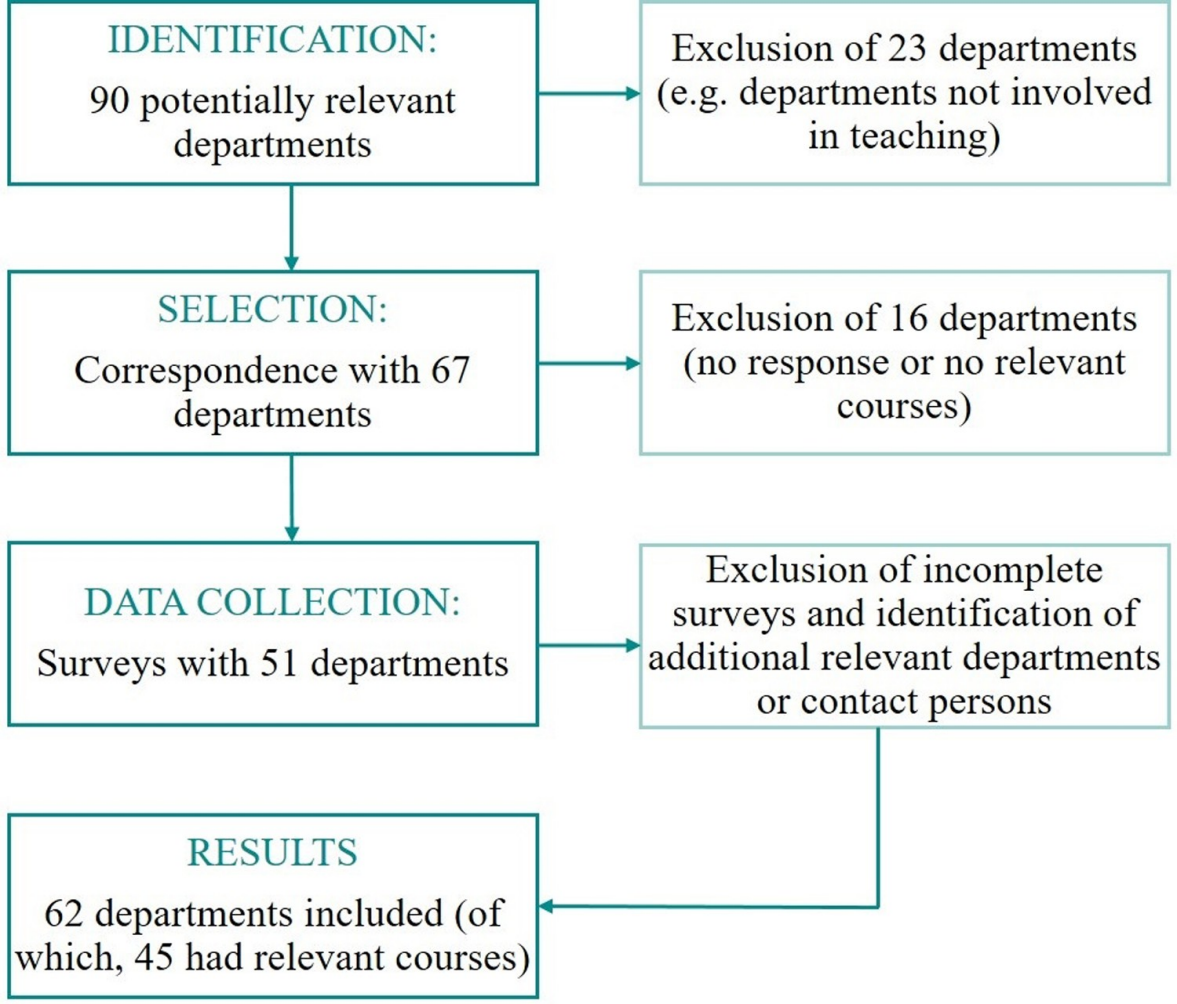
Recruitment of interviewees at the Medical Faculty of Hamburg. Description: All departments, including clinics, administration units and institutes, at the Medical Faculty of Hamburg were identified. The processes for selecting only teaching and relevant departments are shown on the right. The directors were contacted by telephone to obtain the names of the persons responsible for teaching communication-related content. A maximum of three reminders were sent by e-mail or telephone call.

The first survey was distributed to all departments and institutes involved in medical teaching at the Medical Faculty of Hamburg at the end of 2017 and beginning of 2018 (premeasurement). In total, 90 clinics and institutes were identified, 67 were contacted for the survey and teachers from 51 respective clinics and institutes were interviewed in regards to their communication-related courses. The majority of teachers was physicians or psychologists with lectureship at the medical faculty. The number of departments involved had to be adjusted during the interview process, as there was new information about cooperation between the departments. Fig 2 depicts the recruitment of interviewees as already described in Brünahl et al. (2021) [20]. The survey instruments for curricular mapping had already been developed in previous research projects and described in detail in the literature [19–22]. The curricular mapping consisted of three parts: 1) the analysis at the institution, 2) the description of communication courses (13 questions to hour, format etc.), 3) whether any of the 116 NKLM objectives were covered in the course. Participants were asked in a semi-structured interview how much of the course (in %) was spent on communication-related topics to estimate the number of teaching units spent solely on communication. Participants rated the percentage on a five-point scale (10%, 30%, 50%, 70% or 90%), which was then multiplied by the number of teaching units of each course. For example, if a course with four teaching units was estimated to include approximately 30% communication-related content, it was estimated that 1.2 teaching units were spent solely on communication. The aim of the quantitative surveys was to capture the (potential) development of the communication curriculum with a focus on the availability of courses, number of teaching units, coverage of learning objectives, and teaching and testing methods. A second survey of the same participants followed approximately one year later in April 2018 (postmeasurement). This semi-structured interview covered the same topics but focused on changes compared to the first survey.

SPSS Statistics 24 was used to analyze the results and present the descriptive analysis. The survey results were compared to the following two objectives: (1) the curriculum completely covers the learning objectives listed in chapter 14 c of the NKLM [12] and (2) the curriculum consists of at least 300 teaching units (one teaching unit equals 45 minutes), as recommended by the model communication curriculum mentioned above [9, 18, 19].

### 2.3 Qualitative method

#### 2.3.1 SWOT analysis

For the overall goal of identifying supporting factors and challenges for the implementation and optimization of a communication curriculum, a strengths, weaknesses, opportunities, and threats (SWOT) analysis was carried out in September and October 2018. SWOT analyses are a standard tool for analyzing strategies in medical education [28–30]. Representatives of all status groups, including students, midlevel faculty (teachers), professors, and deanery representatives, were asked to discuss the current integration of communication in the curriculum and desired developments. A two-staged process was initiated. First, the respective status groups had the possibility to formulate written results and were given the opportunity to reflect the findings in interviews. Both the student group and the midlevel group participated in the interviews. Every status group decided on the one to five representatives participating in the interviews. The participants assigned each topic they discussed to the category of strengths, weaknesses, opportunities, or threats. The researchers then clustered the results in a table according content similarities. The SWOT analysis helped to inform the strategic planning of the optimization process: enhancing strengths, reducing weaknesses, exploiting opportunities, and minimizing risks. The participants were also eligible for taking part in the group discussions that are described in the following paragraph.

#### 2.3.2 Group discussions

Reflections on both the initial implementation and the optimization process of the communication curriculum were collected with group discussions [31, 32]. The first group discussion took place in July 2017. The project leaders at the Medical Faculty of Hamburg and three other participating faculties and the project coordinators participated. The session was moderated by the principal investigator. The first group discussion aimed to ensure the faculties’ institutional readiness and identify possible challenges facing the project. Strengths, weaknesses, opportunities and threats were also discussed.

Another group discussion was carried out in July 2018. The sessions lasted about one hour. Three psychologists, one medical specialist in psychosomatic medicine and one student worker took part. One psychologist and one sociologist moderated the discussions with the help of an interview guide. The guide contained open-ended questions on the faculty’s strengths and weaknesses related to the implementation of the communication curriculum, institutional readiness, and both planned implementation measures and those that have already been carried out, along with relevant difficulties and facilitating factors [33–35]. In particular, the participants were asked to reflect on the premeasurement results and indicate which factors had contributed to the observed developments. The two moderators recorded and then analyzed the discussions [36].

After the conclusion of the optimization process, a third group interview was conducted in October 2019 with one medical specialist in psychosomatic medicine, two lecturers and a deanery representative. A psychologist moderated the discussion. The interview guide’s subject was the retrospective evaluation of the communication curriculum and assessment of the optimization process. The discussion lasted approximately one and a half hours, and the evaluation was based on discussion transcripts. A researcher analyzed the group interviews by summarizing the findings and examining themes of meaning within the data. Finally, the findings were communicated to the participating institutes in order to comment or revise them.

## 3. Results

### 3.1 The communication curriculum (pre- and postmeasurement)

Analysis of the quantitative data showed that the communication curriculum at baseline was longitudinal and centrally coordinated. There was also a central pool of simulation persons and staff for its management and training. The communication examinations were organized centrally and included multiple-choice questions, open questions, written patient reports, patient presentation and objective structured clinical examinations (OSCEs). No new examinations were introduced during the optimization process. There were no changes in the infrastructural equipment available for communication teaching or the organization. Throughout the entire degree program, communication classes were offered, with the most classes occurring in the second, third and fourth years. In 2019, there were both more courses with communication-related content and more teaching units than in 2017/18 (see Table 1). After the optimization process, all 42 subjects mentioned in the medical licensing regulations integrated communication content.

**Table 1:** Comparison of the integration of communication content in the curriculum between 2017/18 and 2019.

	2017/18	2019
Number of courses with communication-related content	263	272
Total number of teaching units (1 teaching unit = 45 minutes) of courses with communication-related content	772	937
Estimated number of teaching units spent solely on communication	321	338
Proportion of all 42 subjects mentioned in the medical licensing regulation with communication-related content	78.6% (n = 33)	100% (n = 42)
Proportion of communication-related content within the three subjects with the largest % of communication content		
Internal medicine	9.6%	10.6%
Medical psychology and sociology	8.3%	9.5%
Psychosomatics	7.3%	9.2%
Proportion of courses with more than 40% communication content	31.5%	33.8%
Communication embedded in clinical content: Percentage of affirmative answers	100%	100%

All interviewees stated that communication was embedded in clinical content. Table 2 confirms this finding; e.g., it is estimated that 338 teaching units out of 937 total teaching units with communication-related content were spent solely on communication in 2019. In Table 2, the results are compared to the recommended model communication curriculum. The teaching units correspond to the recommendations for concepts, models and general basics (Competence Area 1) and sociodemographic and socioeconomic influencing factors (Competence Area 5). The curriculum more than fulfils the recommended teaching units related to medical communication skills and tasks (Competence Area 2). On the other hand, emotionally challenging situations (Competence Area 3), challenging contexts (Competence Area 4) and other media channels and settings (Competence Area 6) are underrepresented.

**Table 2:** Comparison of the estimated teaching units for the competence areas with those recommended by the model communication curriculum [19].

	2017/18	2019	Model curriculum
	in teaching units (1 teaching unit = 45 minutes)		
**1. Concepts, models and general basics**	21.1	22.1	20
**2. Medical communication skills and tasks**	200.5	212	106
**3. Emotionally challenging situations**	28	29.1	68
**4. Challenging contexts**	19	19.3	35
**5. Sociodemographic and socioeconomic factors**	41.1	43.9	43
**6. Other media channels and settings**	11.4	11.6	28
**Total**	321.1	338	300

The estimates are based on the number of teaching units of a course and the estimated percentage of time spent on communication-related content. The interviewees gave this information on a 5-point scale, with the five categories 0–20%, 20–40%, 40–60%, 60–80% and 80–100%. The category mean (e.g., 20–40% = 30%) is multiplied by the number of teaching units of each course to estimate the teaching units spent solely on communication.

### 3.2 Supporting factors and challenges during the implementation process

#### 3.2.1 SWOT analysis

The SWOT analysis focused on the initial implementation process of the communication curriculum (see Table 3). This revealed that the innovative new curriculum of the model study program has been crucial for integrating communication content into the curriculum since its initial development in 2012. Intense and adequately financed development along with continuous meetings of all institutes was another important structural factor. Financial and other material resources, such as rooms and equipment, were estimated to be sufficiently available. The program’s good organization was another enabling factor for integrating communication content into the curriculum through, for example, central teaching platforms and databases such as Moodle and textbooks. Nevertheless, the participants also mentioned that not all clinical subjects integrated communication content into the curriculum, and cooperation is sometimes tricky since communication is often associated primarily with psychosocial subjects.

**Table 3:** Overview of the main findings of the SWOT analysis regarding the integration of communication in the curriculum.

**STRENGTHS**	**WEAKNESSES**
• The structure and organization of the model study program enable development. • Communication is longitudinally integrated. • Interdisciplinary cooperation has been established from the beginning. • In general, teachers are perceived to be qualified and highly motivated. • Simulation persons are available for practical training. • Communication is relevant for examinations.	• The clinical integration of communication is extendable. • It is perceived that knowledge, interest and willingness to practice among teachers and students is unequal. • There is a need for coordination between different subjects. • The relevance of communication in examinations is somewhat unclear and time delayed.
**OPPORTUNITIES**	**THREATS**
• In general, the relevance of soft skills in medical education is increasing. • Stakeholders can become more strongly involved. • The clinical relevance and relevance for students can be further clarified. • Further didactic options can be explored and used.	• There are limited and competing resources. • Different perceptions of the relevance of communication exist among teachers and students. • It remains unclear whether the transferability of course content to working life is sufficient.

One positive aspect of the curriculum was related to the longitudinal character of the communication curriculum throughout the whole degree program. The introduction to clinical medicine, anamnesis and conversation courses in the first semesters begin to address communication. In the last year of the study–the practical year–the integration of communication is often not as consistent as in the other years because academic hospitals are not well informed about the communication curriculum. “Learning spirals”–methods for independent and individualized learning–were another enabling factor mentioned for integrating communication into the curriculum. The relevance of communication is highlighted by examination formats such as OSCEs throughout the program. However, the relevance of communication in examinations is not entirely clear to teachers, and part of the examined subjects are taught in previous years. Additionally, examiners perceived that they are not always appropriately trained.

The presence of motivated lecturers with competencies in communication and the teaching-learning research conducted at the university were other enabling factors for integrating communication content in the curriculum. Some participants were unaware of the proportions of teaching units on communication offered by subjects within different institutes. The decentralized teaching organization, in which the individual subject areas are independently responsible for implementing communication teaching, was perceived as an impediment for a fully integrated communication curriculum and thus as leading to incomplete coordination and redundancies.

The interviewees attributed immense importance to the faculty members’ attitudes towards and support of the communication curriculum (“top-down”). Notably, in the beginning, there were only a few qualified lecturers, and the development and implementation mainly fell them. Additionally, it was suspected that teachers did not always use their available resources to the full extent. Therefore, participants recognized the need for training lecturers on the learning objectives and the implementation of the content in their teaching.

Furthermore, students can practice both everyday and unusual situations with actors or other students within a pleasant environment to generate feedback and self-reflection. An extensive and professional program for actors is available in which actors are prepared for many roles and varying situations. In addition, extracurricular activities are offered for student tutors (e.g., the summer school in 2015). Challenges include the lack of knowledge and unequal interest and willingness among teachers and students to practice competencies. Not all students are interested, and the same individuals often participate in and profit from training situations.

The interdisciplinary cooperation between groups and regular exchanges between departments contributed to the coordination between both departments and subjects. For example, an anamnesis scheme was jointly developed and distributed as posters and small booklets. Additionally, interdisciplinary and interprofessional teams began teaching courses (e.g., team teaching). Participants mentioned establishing an interdisciplinary working group on clinical examination methods and medical communication as one enabling factor. The strengths associated with this lie mainly in the competences built up through this. Since 2018, the group has been integrated into the annual medical faculty teaching conference, leading to the further institutionalization of communication content. Table 3 provides the main findings of the SWOT analysis.

#### 3.2.2 Group discussions

The group discussions with the stakeholders, including project leaders, teachers, students, and deanery representatives, confirm the SWOT analysis’s major findings, including the integration of communication content into the curriculum. There was a generally positive attitude towards communication teaching, even if not everyone was interested in being actively involved. Many communication teaching competencies were gained, particularly among Master of Medical Education (MME) graduates and current students. Additionally, extensive teaching and communication research areas were mentioned. However, the participants discussed the difficulty of implementing more events because the curriculum is relatively rigidly defined, and the students’ workload limits must be considered. Coordination concerning concrete learning objectives should be further developed and optimized.

In addition to the retrospective group discussions, the project leader summarized that the communication curriculum was developed during the study reform as an integral part of the model study program. This structure enabled concerted support for intensive and financially supported curriculum development. The particularly favorable conditions in terms of institutional readiness, such as established interdisciplinary cooperation, great teaching competence at the faculty and an overall good organization, were helpful.

### 3.3 Supporting factors and challenges during the optimization process

The focus of the optimization process in 2018 and 2019 was on better interlinking the individual subjects and integrating the communication content into the clinical subjects. Furthermore, regular meetings between teachers, information events and meetings for fine-tuning between departments and lecturers were established in the optimization process with the aim of using a common concept. The group discussions in 2019 revealed that the participants viewed the continuous project meetings triggered by the premeasurement as one of the most important factors in optimizing the communication curriculum. Further, the faculty is committed to the project since communication is already a part of the curriculum. The dean and other stakeholders showed enormous support for further developing the communication curriculum. For the group discussions participants, it was crucial that the dean and other stakeholders within the faculty initialized the project with a top-down approach. The coordination group then created opportunities for the participation of other stakeholders, including students and teachers, in the optimization process in line with a bottom-up approach. However, it was mentioned that teachers did not always have the opportunity to implement their ideas and courses because the curriculum is already very extensive. The students’ feedback was mainly positive. The courses on behavior change counseling and motivational interviewing were further developed and optimized based on student feedback, among other things.

Competencies in communication teaching at the faculty, meetings among teachers, continuous coordination processes and the simulation person program also facilitated the optimization process. Barriers included a lack of separate teaching positions for communication and the financing for the curriculum implementation coming from the regular budget. A criticism that arose during the lecturers’ discussions was the desire and the need for supervision and communication training. The implementation of new approaches required restructuring the curriculum, as the curriculum did not easily allow for extensions. In the future, the integration of new content should be planned with a focus on interprofessionality. The perceptions regarding the changes were generally positive, even if not everyone was interested in being actively involved. The project leader summarized that the support and communication curriculum at baseline enabled development during the implementation process.

## 4. Discussion and conclusion

### 4.1 Discussion

The study results show that there is a comprehensive, coherent and longitudinal communication curriculum integrated into the main curriculum at the Medical Faculty of Hamburg. Additional differentiated structures were developed during the project and throughout the optimization process, and communication is now a more prominent part of the faculty’s self-image than it was before the project. The optimization process in 2018 and 2019 was successful due to the dean’s support for the project and the excellent baseline conditions, such as research conducted at the university [37]. Another enabling factor was the revival of monthly working group meetings. Similar to what has been found in other publications, a lack of awareness in the form of negative attitudes towards communication teaching, disinterest and a lack of support were regularly mentioned as hurdles [19, 38–40]. In contrast, however, a supportive deanery, motivated lecturers and committed students actively helped shape the implementation process through this top-down and bottom-up approach.

The results show that one great benefit of the new curricula of model programs is the integration of relevant competencies and practical skills from the beginning. Developing modern curricula requires much consideration but enables a high degree of integration of relevant topics such as communication, as shown at the Medical Faculty of Hamburg. Curricular mapping is essential for identifying the strengths and weaknesses of a curriculum. One strong advantage of this project’s approach is that all different groups were included since all stakeholders were crucial for the development process. The project sought to combine both top-down and bottom-up approaches. Decisions for the development of the curriculum were primarily top-down, ensuring efficiency and supervision. The dean’s support paved the way for meetings and for curriculum adaptation. On the other hand, it is also crucial to boost bottom-up capacity through the extensive integration and involvement of all stakeholders. Lecturers and students were motivated to participate and to improve the teaching at the faculty.

The comparison to the NKLM showed that some competences are better covered than others. These shortcomings have to be identified, e.g., with a curricular mapping, communicated and discussed. Then the curriculum or existing courses should be revised accordingly. Although the optimization process resulted in an improved curriculum, development is ongoing and must provide for adaptations to new demands and needs. Simultaneously, it is necessary to ensure that taught material is understood and can be implemented by medical students. Practical examination formats, such as OSCE, have proven their usefulness in this context [29, 38, 41]. The relevance of teaching and assessing competencies in medical studies is recognized and highlighted by recent policies and initiatives [9, 12, 19]. One next step is to integrate practical examination formats in national licensing examinations to ensure nationwide quality standards and, thereby, improve outcomes for patients, healthcare staff, and society [9].

Limitations of this study include that the analysis focuses on one curriculum at one medical faculty only, and the generalizability of the findings is therefore limited. However, it is a fascinating example that shows that communication content can be integrated into a curriculum within a relatively short period of time. The model program had different framework conditions than traditional courses of study in the sense that the model character allows for more scope for design and innovation [25, 42]. Further limitations include that the analysis does not capture the level of taught competency [41] and that no distinction was made between the explicit and implicit teaching of the learning objectives [43]. Both would have significantly expanded the survey’s scope and reduced the survey instruments’ practicability. The number of teaching units spent solely on communication was based on estimations since the content was integrated into other subjects.

### 4.2 Conclusion

The importance of communication competences for doctors is recognized worldwide, particularly at the Medical Faculty of Hamburg. A comprehensive, coherent and longitudinal communication curriculum was integrated into the curriculum and optimized during the project through the cooperation of different status groups. In this way, the medical faculty set the course for a successful start to the students’ careers, as future doctors can benefit from learning communication skills during their studies.

### 4.3 Practice implications

Our study shows that there are many prerequisites for the successful implementation of communication curricula. These factors may differ from university to university and across countries depending on the structural conditions and maturity of the curricula. Before implementing a project similar to ours, the baseline conditions should be evaluated (premeasurement). Quantitative surveys help identify developments during the project phase (pre- and postmeasurement), and qualitative discussions with all stakeholders are necessary to identify specific challenges and ways to overcome these challenges.

Based on our results, it is evident that whenever possible, communication content should be integrated into the development of a curriculum from the beginning. Second, the dean needs to support such development and ensure the support of faculties’ employees. Third, the cooperation of all stakeholders and regular working group meetings are crucial. Last, the development of (communication) curricula should be ongoing.
